# Contesting liberal-colonial citizenship: the planetary model of citizenship and the struggle for the ‘right to shelter’

**DOI:** 10.3389/fsoc.2025.1520611

**Published:** 2025-07-09

**Authors:** Catharina Peeck-Ho, Mathias Bös

**Affiliations:** ^1^Institute of Social Sciences, Carl von Ossietzky University, Oldenburg, Germany; ^2^Institute of Sociology, Leibniz University Hanover, Hanover, Germany

**Keywords:** planetary citizenship, liberal-colonial citizenship, sanctuary cities, migration, United States, right to shelter law, Massachusetts

## Abstract

Anti-immigrant mobilization has reached a new peak with the rise of right-wing neo-fascist movements and many problems in contemporary societies are discursively linked to immigration. These developments pose new challenges to the ongoing struggle for immigrants’ rights, as current discourses on so-called “sanctuary cities” in the United States demonstrate. The article makes the case that these phenomena are connected to different knowledge orders about citizenship and its underlying principles. While the liberal nation-state is based on the idea of the equality and national sovereignty, new social movements have fundamentally problematized global social inequalities and injustices. Their visions are not only about equality between humans, but include a different understanding of society’s relationship with nature. In this article, we argue that the normative foundations and knowledge orders associated with these issues are accompanied by different—and sometimes incompatible—models of citizenship, which can be typified as ‘liberal-colonial citizenship’ and ‘planetary citizenship’. They imply different notions of belonging and social justice and emphasize different forms of rights (e.g., citizenship rights vs. human rights). An analysis of current discourses on the so-called ‘right to shelter’ law in Massachusetts shows how different models of citizenship are applied to legitimize political claims, suggesting either an inclusive model for dealing with immigration or excluding non-citizens. The paper shows how the legal and administrative inclusion of immigrants reflects contested knowledge orders about the content and normative basis of citizenship within these struggles.

## 1 Introduction

Against a backdrop of rising nativism (Duyvendak et al., 2022), immigration has become a key field of political polarization in Western democracies. Immigrants are particularly affected because they usually do not have the same legal protections as citizens. As a result, the legitimacy of rights claims and thus the nexus between migration and citizenship are highly contested. To what extent should immigrants be able to exercise social or political rights? Are they considered valuable parts of society or outsiders? These issues are discussed here as expressions of different knowledge orders surrounding citizenship. They provide an example of how social discourses in the field involve fundamentally different and sometimes contradictory ideas about membership and belonging. To better understand these dynamics as examples of the political debate around the migration-citizenship nexus, the article will focus on the normative foundations and knowledge orders, associating them with two models of citizenship: ‘liberal-colonial citizenship’ and ‘planetary citizenship’. The connections between immigration, citizenship, and belonging are linked to different understandings of which social groups and individuals can legitimately claim citizenship rights, particularly social rights.

The right to shelter is an example of how knowledge about citizenship and immigration is contested, and of which are the legal and social consequences of these struggles. This case illustrates how various forms of knowledge about citizenship are interwoven in current discourses and associated with different—and occasionally conflicting—normative orders. Given the federal system that characterizes governance in the United States, these processes invariably involve references to different scales of citizenship and reflect conflicts between local and federal policy. Political debates reflect the ongoing negotiations among actors at different levels, providing material to consider how the migration-citizenship nexus is negotiated between the local level, states, and the federal government, as well as how this is linked to competing constructions of citizenship. The article’s primary focus is on the second aspect. Consequently, the analysis of local discourses on citizenship and belonging is at the core of this investigation. Because it applies to both citizens and non-citizens, right to shelter is particularly well suited to illustrate how legitimate access to rights is connected to constructions of belonging. Analysing contemporary discourses on liberal-colonial and planetary citizenship as competing models provides a foundation for understanding these nuances. By confronting these two knowledge orders of citizenship, we analyse how different normative orders are embedded in discourses on immigration, determining who is welcomed and can legitimately claim rights on this basis. The underlying research question is therefore: How do current socio-political debates on sanctuary laws reflect competing knowledge orders about citizenship and related normative understandings of migrants’ belonging and rights? Answering this question can help us better understand current debates over sanctuary cities. It also sheds light on broader conflicts concerning the relationship between migration and citizenship in U. S. society. In order to address the issue of how citizenship is transformed and negotiated in relation to immigration, the sociology of knowledge approach to discourse analysis (SKAD) (Keller, 2005, 2012) was employed. This analysis examined the discourses on the right to shelter in Greater Boston in the period from March 2023 to June 2024.

The article begins with a discussion and outline of the two competing conceptions of citizenship: the liberal-colonial model and the planetary model. These two conceptions are subject positions in the political discourse embedded in different orders of knowledge. These competing orders of knowledge relate to the nature of democracy, the basis of political membership and the relationship between rights and duties. The issue has become a real feature of social struggles over citizenship in late modern society. In the second part, we examine the struggles over these models of citizenship. Using the discourses on the right to shelter as a form of sanctuary legislation in the United States, we show that migration became one of the key areas of these struggles and how they are linked to particular models of citizenship which relate to the two proposed models. The conclusion considers how these findings can contribute to an understanding of the nexus between migration and citizenship against the backdrop of current tendencies of social and political polarization.

## 2 Competing models of citizenship: Liberal-colonial citizenship and planetary citizenship

In order to understand current debates in the field of migration and citizenship it is useful to distinguish between two different ideal-type models of citizenship associated with competing orders of knowledge. Drawing on theories of reflexive modernization (Beck, 1993; Beck et al., 1996), the authors explain that social knowledge is becoming increasingly pluralized, leading to new pressures for decision-making in society. While the logic of industrial modernity was essentially characterized by the idea of unambiguity, different and sometimes competing orders of knowledge are increasingly visible. They have varying sources of legitimacy and ensure the coexistence of different bodies of knowledge, which in turn are socially reflected in diverse options for shaping life. Accordingly, one of the central assumptions of reflexive modernization is that the increase in knowledge does not lead to more clarity in decision making. Rather, it produces new needs to decide certain questions socially and individually. This applies to all kinds of issues, including family and family forms, work, and social diversity, the relationship between humans and nature (Bonß and Lau, 2004).

Current discourses on citizenship and belonging can be regarded as a mode of negotiating knowledge in late modernity and an effect of competing orders of knowledge that produce divergent notions of who can be considered legitimate claimants and who is excluded from these processes. With the concept of planetary citizenship, we aim to rethink familiar notions of global citizenship (Isin and Nyers, 2014). In recent years, sociologists have argued that introducing the planetary into the debate can broaden our perspective on current and future social developments (Block, 2022). In particular, it allows us to rethink the relationship between society and nature in late modernity, as technological developments, climate change, and environmental issues come into focus of analysing social change. The perspective of planetary citizenship raises questions about the formation of political subjectivities in this context and related attributions of responsibility which have an impact on various social fields, among them migration, citizenship and belonging. The ways in which nature and society are or are not discursively addressed are linked to notions of legitimate membership and access to rights. For instance, climate change is increasingly recognized as a driving force behind migration (Schraven, 2023, 629). This raises new questions regarding the legitimacy of migration decisions. Thus, the claims of political actors need to be understood against the backdrop of changing knowledge orders that legitimize or delegitimize access to rights on different grounds. Within these negotiations different approaches towards belonging, inclusion, and exclusion can be identified. While some focus on the relatively homogeneous concept of nation-state, others propose global solutions to address inequalities and social problems. Accordingly, several ideal types of citizenship can be identified, including those of liberal-colonial citizenship and planetary citizenship, on which this paper focuses^1^. These concepts are used because it is believed that they can contribute to a more nuanced understanding of the significant polarisation observed in contemporary societies.

The notion of liberal-colonial citizenship indicates the continuities of colonialism within the modern paradigm of liberal citizenship as opposed to the republican ideal (Bös and Peeck-Ho, 2025). Historically, the modern conception of citizenship emerged during the revolutionary period of nation-states formation, such as in the case of American Revolution (1765–1783) and the French Revolution (1789–1799) (Steinmetz, 2014). The revolutionary formation of citizenship in emerging nations incorporated colonialist economic and political ideas (e.g., USA; Smith, 1997), so that many of today’s nation-states ended up incorporating historically determined inequalities and lines of conflict, such as conflicts over the civic inclusion of Indigenous or enslaved peoples, or in the case of racialised immigrant minorities. This colonial-national model of citizenship shifted to a liberal-colonial model after the Second World War. According to this model, two aspects were central to the relationship between the rulers and the ruled: (1) the provision of basic services to all citizens, especially health care and social security, and (2) the facilitation of inclusion in family, neighbourhood, work and social life through citizenship (Marshall, 1950). Despite attempts to ensure full citizenship and formally equal rights to national citizens, *de facto* inequalities within nation-states borders are observed (Parsons, 1965). The resulting economic and political coloniality of citizenship is reflected in its persistence in perpetuating global inequalities, and in global political and economic institution framed as modern but deeply rooted in colonial exclusion (Boatcã and Roth, 2016). In addition to the legal and physical exclusion of women, slaves, and the dispossessed, non-European, non-white, and non-Western populations were excluded from civil, political, social, and cultural rights. These mechanisms persist to the present. Implying the idea that all nations are equal and sovereign, liberal-colonial citizenship neglects colonial continuities and environmental inequalities between nation-states. Citizenship, functions as inherited ‘property’ that excludes non-citizens from wealth and opportunity. It is maintained by ascriptive means such as birth in *jus soli* or descent in *jus sanguinis* systems (Shachar, 2021 [2009]).

In contrast to the liberal colonial model of citizenship and the knowledge systems associated with it, planetary citizenship can be viewed as the result and basis of new social movement discourse since the 1960s. It includes cosmopolitanism, embraces social diversity and supports state regulation to address social challenges such as inequality, discrimination, environmental degradation or climate change. In contrast to the liberal-colonial citizenship, planetary citizenship brings forward social and environmental justice on a global scale, arguing against the exclusive character of liberal colonial citizenship (Brandzel, 2022). As a result of the struggles of new social movements and the recognition that society increasingly has to deal with the side effects of modernity (Beck, 2012 [1986]), its proponents claim that citizenship need to be based not only on birthrights, but also on rights based on historical injustices, global power relations and human rights. It thus encompasses fundamental aspects and issues that have been previously discussed under the term global citizenship, such as the reference to political subjectivities claiming rights on the grounds of human rights debates and the reconceptualization of citizenship as a performative category (Isin and Nyers, 2014). However, it directs the discourse with greater emphasis toward the associated attributions of responsibility and the manner in which they serve as the foundation for normative orders surrounding citizenship. Concretely, this entails a broadening of the range of legitimate reasons for fleeing, with war being just one such example. Climate and environmental refugees are increasingly becoming central to the discourse. To characterize and examine ‘planetary citizenship’, two central aspects can be distinguished. First, there are normative references to human rights and social justice. These references are linked to critiques of colonial relations and global inequalities. For several years, representatives of citizenship studies have discussed these references under the heading of ‘global citizenship’. These are now being linked to new questions about the relationship between humans and nature in the context of planetary citizenship as environmental protection and climate change issues become increasingly urgent. On the basis of this knowledge order, current crises are addressed as [1] side effects of liberal colonial formations of capitalist society and [2] as interrelated phenomena, for example when links are made between climate change, growing inequalities and social conflict (Fraser, 2022). In addition, the concept of planetary citizenship is predicated [3] on the notion of the city as a metaphor for civic engagement and democratic deliberation, both on a local and global scale (McNevin, 2022). For the field of migration and citizenship, such a view means, for example, that migration as a consequence of climate change is becoming a focus of activism and discourse (Koubi et al., 2021; Schraven, 2023). The perspective of planetary citizenship also raises new questions about access to rights, for example when environmental rights are discussed as a precondition for social rights and equality. If, for example, access to clean drinking water is restricted to certain population groups, the relationship of society to natural resources needs to be reconsidered, as can be witnessed, for example, in current debates on environmental rights in Latin America (CIDH and OEA, 2023). Planetary citizenship is seen as an order of knowledge and ideal type that engages with the dynamics of creating inequalities within social structures and connects them to the ways in which society relates to social justice, nature and its resources.

The current struggles for citizenship are therefore closely linked to the historical developments of recent decades and even centuries. Based on different orders of knowledge, liberal colonial citizenship and planetary citizenship imply different views on what constitutes proper citizenship and who can legitimately claim access to rights. As a result, the two models imply different ideas of belonging, of inclusion and exclusion, equality and inequality in society. The discourses on the significance and the assessments of immigration as an opportunity or threat to society contained therein point to different ideas of legitimate belonging that are associated with these different models of citizenship. An analysis of the legislative framework pertaining to so-called ‘sanctuary legislations’ in the United States can offer valuable insights.

## 3 Struggles on immigration in the United States: The Massachusetts right to shelter law

Sanctuary policies for immigrants emerged in the United States in the 1980s in response to the treatment of refugees from Central America at the time (Collingwood and Gonzalez O'Brien, 2019: 16–18). Since the federal government had no interest in accepting refugees from the civil wars in El Salvador, Nicaragua, and Guatemala for political reasons, the acceptance rates were very low. For example, only 3% of Salvadoran asylum claims and 2% for asylum claims made by refugees from Guatemala were accepted (Bau, 1994: 50). Most refugees were rejected. In this context, mostly church-based initiatives and networks were formed to support these refugees and their claims. They contributed to the creation of laws and administrative guidelines at the municipal level, which are now discussed under the term ‘sanctuary’ by researchers from different disciplines (e.g.: Ridgley, 2010; Villazor, 2010; Lippert and Rehaag, 2013; Mancina, 2016; Delgado, 2018). Although a single definition of the term is not existing (Delgado, 2017), many authors emphasize the limitation of cooperation with national authorities, especially Immigration and Customs Enforcement (ICE) (Wong, 2017; Collingwood and Gonzalez O'Brien, 2019). Some authors stress the importance of support networks for immigrants as part of the sanctuary movement (Vitiello, 2022; Bruhn, 2023). Others regard it primarily as a form of governance of populations without citizenship status (Mancina, 2016). This is for example linked to the introduction of local identity documents, so-called “City I. D. cards” in cities like New Haven, San Francisco, Chicago or New York since 2007.

With the decline in immigration in the 1990s and the changes in security policy since September 11, 2001, conditions have changed. Since the founding of the New Sanctuary Movement in 2007, this commitment has focused primarily on illegalized migrants who live in the country for longer periods (Bauder, 2016: 3), especially the so-called ‘DREAMers’ who came to the U. S. as children (Ortega et al., 2019). With the presidency of Donald Trump, the discourse on the issue again has transformed again. While migration had been marked as a threat to public security already after 9/11 (Weber, 2013), this has been fortified during Trumps presidency (Peeck-Ho, 2021) and by its supporters in the Republican party. An important discursive event that made these changes possible in the first place was the death of Kathryn Steinle, who was shot and killed by an undocumented migrant on a San Francisco pier in July 2015 (Collingwood and Gonzalez O'Brien, 2019: 16). It significantly shaped the debate around sanctuary cities and has been used by many advocates of a more restrictive approach to migrants to portray their presence as a security problem (Peeck-Ho, 2021).

Section 30 of Chapter 23B of the General Laws of Massachusetts was enacted in 1983 and is the focus of this article. It guarantees families and pregnant women the right to shelter regardless of immigration status. The right to shelter is a family-based right. Having children is an integral part of eligibility. Being a law on the state level which does not differentiate between citizens and non-citizens, ‘right to shelter’ can be discussed as a form of sanctuary legislation which is particularly under attack. Against the backdrop of a lack of housing, especially in the Boston area, it is increasingly difficult to find affordable housing, especially for low- and middle-income families. In this respect, the discourses on the ‘right to shelter’ represent a development in which different social problems are linked and placed in the framework of migration. The relationship between migration and resource scarcity represents a pivotal point of contention in contemporary discourses on citizenship in the United States. Those who oppose the ‘right to shelter’ have concentrated their criticism on the number of immigrants who are perceived as placing an excessive burden on the shelter system. This discourse gives rise to a variety of interpretations of inclusion and exclusion, which have tangible consequences for population groups living in the country without permanent residence status. The aforementioned conflicts provide an opportunity for a discussion of the ideal types of planetary citizenship and liberal colonial citizenship, which are believed to underlie the migration-citizenship nexus in the present context.

## 4 Data and methods

The analysis is based on methods and principles of the sociology of knowledge approach to discourse analysis^2^ (SKAD) (Keller, 2005, 2012). In a two-stage process, the political and media discourse surrounding the field in Greater Boston in the period from March 2023 to June 2024 was first tracked using local daily newspapers such as the Boston Herald (26 articles) and the Boston Globe (30 articles), publications by the MassINC Commonwealth Beacon think tank and publications and speeches by local politicians on the topic (18 publications, 1 podcast). Subsequently, 20 documents were selected for detailed analysis, corresponding with the criteria of grounded theory, especially the concept of theoretical saturation (Glaser and Strauss, 2006 (1967). These documents include newspaper articles, documents from NGOs, and documents and information published by the cities themselves. The documents show how these discourses are structured, which interpretations of citizenship are involved, and how they are legitimized, using methodological tools from the framework of the sociology of knowledge approach of discourse analysis (Keller, 2005, 2011, 2012). The documents were initially subjected to a systematic evaluation in order to facilitate a structured representation of the associated discourses of belonging. A phenomenon structure (Keller, 2005: 242–246) identifies the central dimensions that are negotiated and the different forms of occupying discursive positions. In the second step, the subject positions are presented in relation to the competing knowledge systems about citizenship that are the focus of this study. In light of the aforementioned, we posit that an analysis of contemporary discourses pertaining to competing models of citizenship provides a foundation for comprehending the nuances of contemporary political conflicts on citizenship and membership, particularly in the context of immigration.

One insight that can be derived from discourse analysis is that language is linked to power (e.g., Hall, 1997; Keller, 2005, pp. 134–140). This is reflected in the knowledge order, which can be partially addressed by analyzing how elements are named within a discourse. To analyse the migration and citizenship nexus from a knowledge-centred perspective, it is useful to consider the terminology employed. The vocabulary chosen can provide significant insight into the legitimization or delegitimization of access to rights in relation to categories associated with belonging. In order to approach the question of how citizenship is transformed and negotiated in this context and to be able to make statements about the competing models discussed above, the constructions of belonging and the dimensions mentioned will be examined. This will entail an analysis of the discourses on the right to shelter to identify the descriptions of the self and the other, the ideas of inclusion and exclusion, and the normative foundations for access to rights that they contain. The period during which public discourse on sanctuary cities was analysed corresponds to a time when public discourse in Boston was predominantly centred on the city itself. Pressure from the federal government had experienced a certain degree of attenuation following the initial attempts of the Trump administration to compel cities to adhere to federal regulations (Bill H.1807, 191st; 2019–2020). Subsequent to 2021, during the Biden administration, the state of Massachusetts made persistent efforts to withdraw funding from so-called ‘sanctuary cities’. In August 2023, the Massachusetts Governor, Maura Healey, declared a ‘state of emergency’ in a letter to the Department of Homeland Security due to overcrowding in her state’s shelter system. Massachusetts is the only State with an Emergency Housing Assistance Program which guarantees families and pregnant women a right to shelter regardless of their residency status. Implemented in the 1980s, it was considered a successful form of sanctuary legislation and a step toward including immigrants at the city level for decades. Furthermore, it provided a strategy to support undocumented immigrants after their arrival. However, by 2023, the shelter system was overwhelmed due to the high number of incoming immigrants. Healey’s letter states that the ability to house people in emergency shelters is limited due to various factors, including a lack of affordable housing and federal immigration policy: *‘The need for action is urgent’* (Healey, 2023). In the months that followed, the law became the centre of a heated public debate about the limits of granting rights to immigrants in the State and its impact on society. By doing so, different social phenomena were linked discursively. For example, migration and so-called ‘sanctuary legislation’ were connected to fields such as economic inequality, housing policy and public safety. Proposals for dealing with the problem should be interpreted in light of this context: Among the voices arguing that the right to shelter should be limited was State Representative Peter Durant, for example. He filed a petition stating that the Emergency Housing Assistance Program should be applied to U. S. citizens solely (Durant, 2023). In November of the same year Healey chose a different option and limited the right to shelter to 7,500 families regardless of citizenship status (Mass.gov, 2023). Finally, in 28.12.2024, Gov. Maura Healey pledged the cost of state-run shelters would decrease, and that Massachusetts is not a ‘sanctuary State’.

## 5 Who’s entitled to the right to shelter?

The discourse analysis reveals the existence of two primary positions, which can be distinguished based on their respective premises. Both perspectives concur that the prevailing circumstances are untenable. However, while some argue that it is imperative to provide shelter to all residents, including immigrants, and consequently advocate for augmented resources, others contend that the core issue pertains to the groups entitled to shelter. In essence, while some perceive a right to shelter for all individuals residing in Massachusetts, others underscore the distinction between citizens and non-citizens. These two positions are associated with markedly disparate notions about the role of immigration in and the overarching character of American society. Consequently, they espouse totally different positionings with respect to the citizen-migration nexus. The following paragraphs highlight key aspects of these discourses. First, it examines the normative foundations for legitimate access to the ‘right to shelter’. Next, migration and its various effects are discussed. Third, citizenship is discussed in terms of inclusion and exclusion.

### 5.1 Normative foundations of belonging and citizenship

A first issue that emerges from the debate on the topic is the naming of the groups to whom the right to shelter is addressed. While proponents of an inclusive concept typically emphasize that it concerns all residents of the state, or highlight the need for assistance of immigrants, opponents of this policy often cite citizenship status. In an interview with the Republican state representative Peter Durant, radio host Dan Rea states:

“*Here’s the concern that I think most normal citizens in Massachusetts have: And the concern is we have tremendous influx of—I use the word illegals, the governor now tries to use the word newcomers, and some use the word migrants, but people who have come to our state and take advantage of the right to shelter law*” (Rea, 2024)

The speaker employs the conventional lines of argumentation characteristic of right-wing movements during the interview process. This entails an implicit differentiation between national citizens and an unidentified collective that ostensibly fails to meet the aforementioned criterion. In terms of justifying the necessity of restricting the right to shelter, the concepts to talk about the population groups eligible for support, is notable. In this interview and elsewhere, the speaker refers to the people in question as ‘illegals’, thereby distancing himself from the positions of the Healey administration. For him, the distinction between ‘citizens’ and ‘non-citizens’ represents a fundamental division that justifies the claim to certain rights. The notion of liberal-colonial citizenship is represented here by legitimizing unequal access to services by limiting legitimacy to citizens. Regardless of specific needs, immigrants are assumed to have an illegitimate advantage when the right to shelter is applied to them. This also calls into question international conventions on the rights of the child, such as the right to privacy (Art. 16) and the protection of refugee children (Art. 22) (United Nations, 1989). Given that the right to shelter is limited to families with children and pregnant women, statements that generalize migrants in order to de-legitimize their claims to adequate housing as a social right imply a fundamentally different interpretation of legitimate inequalities. Much like colonial subjects who were historically denied the same rights as citizens, immigrants are treated as second-class citizens.

It has already been argued that citizenship remains a critical factor in creating and legitimizing inequalities globally and domestically. The denial of basic social rights to immigrants here perpetuates these tendencies. This position is fundamentally different from those which centres on families as the most important category to define who has a right to be sheltered. In contrast for example the Executive Office of Housing and Liveable Communities states that:

“*The Healey-Driscoll Administration’s goal to ensure that shelter for families is temporary, supportive, and non-recurring. However, the emergency assistance (EA) program continues to see demand for shelter beyond the fiscal and operational capacity of the system. The administration is making these changes so that the program can continue to exist within current and future fiscal constraints while redirecting families into more stable housing options outside of the EA system*.” (Executive Office of Housing and Livable Communities, 2024)

The citizenship status of these families is not a significant factor in this and other documents by the Healey administration. Interestingly, little is said about the normative foundations of these considerations. Many articles point out that the right to shelter should be guaranteed, but the question of why this is so is rarely addressed. Statements of this nature can be found primarily in the publications of immigrant advocacy organizations, which emphasize human equality and, to a certain extent, the contribution of immigrants to US democracy. In this context, human rights serve as a fundamental basis for activism and, consequently, also for access to social services. The Massachusetts Immigrant & Refugee Advocacy Coalition (MIRA), which administers the Immigrant Assistance Services Program (IAS) for immigrants in temporary accommodation, documents its vision as follows:

“*Our vision is a Commonwealth—and a nation—where all can thrive, no matter where they came from or how they got here, and all can fully participate in their community’s social, economic, and civic life*.” (Massachusetts Immigrant and Refugee Advocacy Coalition (MIRA), 2022)

This is associated with values such as inclusion, collaboration, capacity building and a general focus on migrants and refugees. The current campaign “Protecting Our Immigrant Communities” (POIC)^3^ points to the ways this is related to legal considerations as it refers to the principle of equal legal treatment regardless of immigration status (Massachusetts Immigrant and Refugee Advocacy Coalition (MIRA), 2025).

Human rights are therefore an important basis for these interpretations of the right to shelter. References to responsibility remain implicit. However, they seem to be included indirectly in the statements of political actors who emphasize the comparatively good living conditions in Massachusetts compared to the countries from which people migrate. In her “state of emergency” letter to Alejandro Mayorkas, Secretary of Homeland Security, Healey explains for example:

“*Many of these families are migrants to Massachusetts, drawn here because we are and proudly have been a beacon for those in need. […] These families require help to obtain housing, food, medical care, education, diapers, and infant formula. Some a fleeing imminent threat of violence. They all have one thing in common. They are in danger of going without the most basic of human rights in one of the most prosperous places on earth: the ability to lay their heads down in a safe place every night with a roof over their heads and with access to fundamental human necessities. They have called us to help give them shelter and the ability to work*.” (Healey, 2023)

Healey implicitly refers to precarious living conditions, scarcity of resources, and conflict as causes of flight and migration. Even if it is not directly stated that this leads to a responsibility to accept refugees, the conclusion seems plausible. The idea that respect for human rights is a principle of political action suggests that it is based on a sense of citizenship, the scope of which is not so much the nation-state as such, but humanity, which has a common responsibility for one another and for solving crises. The idea of planetary citizenship is thus inherent in these statements, even if it is not explicitly formulated as humanity’s responsibility for one another and for the environment that surrounds them.

Right to shelter as a family-based right is seldom questioned within the debate. One exemption is the line of argument brought forward by Senator Ryan Fattman in October 2023. He filed a petition to limit shelter to Massachusetts residents who are U. S. citizens. In a speech in the General Court, Fattman argues that initially the law was intended to support Massachusetts residents and involved a paragraph which defined residency and required a proof of residency. In 1991 this part was removed, an action that Fattman directly connects to the current “crisis.” According to him, the main problem lies in the production of new inequalities by the law which become particularly visible in the context of increased immigration to the state:

“*And one of the frustrations for me in my office is that we were contacted by my brother, who is overseas serving our nation. He has a soldier whose father is homeless. And in this wonderful right to shelter principle state that we have, we have been unable to find his father shelter who is a veteran. And today he is living out of his car, So, when we start throwing—as we did in the last supplementary budget—70 million dollars for increased funding to emergency shelters, 37 million dollars for transition into such shelters for migrants, 350 million more dollars in this budget for emergency shelter systems and we can’t find a place for a Vietnam veteran who served our nation, who is a Massachusetts resident. That’s a really significant problem*.” (Facebook, 2023)

In contrast to positions that emphasize the vulnerability of children and families, Fattman asserts that the right to shelter should be extended to individuals who reside in the country and have served it in the military. The idea of deservingness, whereby the veteran is regarded as a person who has served the country and thus has earned the right to support can be seen as a continuity with patterns of argumentation of liberal-colonial citizenship that prioritizes political membership as the status of citizenship and add the relationship to the nation-state—in this case expressed through military service—as the basis of rights.

It shows that in times of transnationalization and globalization, the idea of the nation-state, as it was established in the nineteenth century and produced in the sense of an imagined community of destiny [Anderson, 2016 (1983)], is still so plausible that international agreements take a back seat to it. Historically the prospect of colonial-liberal citizenship remained contingent upon the gradual integration of diverse groups into the nation-state society and the democratic process. Marshall’s (1950) model, in particular, underscores the potential for this evolution, particularly in relation to the working class, which, according to this model, would be transformed into citizens through the civil right to education. The development of social ties, or ligatures, was seen as a key factor in fostering national solidarity (Dahrendorf, 1957).

Since the early 1990s, colonial-liberal citizenship has been increasingly unable to ensure stable employment histories and to provide a sufficient standard of living, including through transfer payments, thereby failing to uphold one of the central promises of liberal citizenship. Migrant families, on the other hand, seem to be unfairly favoured, an argument that appears in many of the statements made by opponents of assistance to migrant families. This can be interpreted as an indication of how the different perspectives on the right to shelter are normatively legitimized. In addition to the demand to limit it to families who have U. S. citizenship, positions are also visible in the discourse that distinguish even more clearly between those who identify human rights and those who emphasize deservingness and commitment to the country in certain services (such as the military) as a basis for access to social rights. Fattman’s statement is no exception to this phenomenon: right-wing commentators, such as Howard Carr who regularly publishes in the Boston Herald, repeatedly point to the alleged lack of loyalty of immigrants to America (The Boston Herald, 2024).

### 5.2 Immigration as a chance vs. immigration as a threat

A second aspect of the argumentation can be identified in the way the issue of migration is addressed. The current circumstances, especially in the Boston area, are commonly perceived as a crisis. Nevertheless, there is significant heterogeneity in the proposed solutions regarding the role of migration in U. S. society. While some proponents of the cessation of migration have advocated for the implementation of more stringent border crossing regulations at the southern border of the United States, others are committed to protecting migrants, perceiving this as an obligation the United States has towards individuals in need of protection. Immigration is thus perceived as either a threat to social cohesion, economic prosperity, and the security of the population or as an opportunity for local communities to gain new economic and social impetus through diversity. In an opinion piece published in the Boston Herald, right-leaning columnist Howard Carr presents the initial perspective, which he outlines in stark terms. Referring to the plans of opening a new shelter in the town of Norfolk, M. A. he claims:

“*It’s not just Norfolk that’s going to be destabilized by the sudden arrival of 450 illegal aliens who don’t speak English, don’t work, mostly feel entitled and have absolutely no regard for any American laws, even the ones that are supposed to protect children. Norfolk shares a regional middle and high schools with Plainville and Wrentham. Those towns’ property values are about to take a dip as well as the schools are deluged with non-English speaking students. Plus, the closed jail-turned-flophouse is located on the Walpole town line. So, Walpole is also going to have its first responders overwhelmed answering endless both frivolous and criminal 911 calls*.” (The Boston Herald, 2024)

In addition to the economic concerns previously outlined by the author, he repeatedly suggests that the arrival of immigrants increases crime and violence. For example, he claims that immigrants purchase vehicles with the assistance of state-sponsored programs, yet they would neither secure insurance nor obtain a driver’s license. This perpetuates a homogenizing view of immigrants in the emergency assistance program. The assumption that they will not adhere to the established rules is generalized and is neither substantiated nor verified with regard to the prevalence of certain phenomena. Therefore, the fundamental conflict appears to be between citizens who can legitimately claim rights and adhere to established rules, and migrants who do not follow these rules or are even unaware of them. In sum, the presented scenario is one of considerable dismay It has been designed to provoke feelings of apprehension regarding the prospect of foreign incursion.

In contemporary discourse, tendencies that bear resemblance colonial stereotypes can sometimes be discerned in the rationales employed to justify the treatment of migrants. As the commentary shows, the attributions under scrutiny bear a striking resemblance to categories that have historically been used to justify the unequal treatment of colonial subjects, particularly characterizations as undisciplined, uneducated, and potentially dangerous.

It is important to emphasize that the position presented here constitutes a pronounced illustration of homogenizing and constructing some parts of the population as ‘others’ thereby legitimizing the restriction of access to rights based on this categorization. A significant proportion of the dissenting voices that are calling for restrictions on the right to shelter are moderate. However, this should not be overlooked. The Boston Herald, one of the two dominant daily newspapers in the Boston metropolitan area, does not cater to a niche audience within right-wing extremist circles. The presence of such views in a prominent daily newspaper underscores the potential for extremist ideologies to permeate the mainstream by means of othering.

In contrast to these views, positions which defend right to shelter frame diversity as an opportunity for communities. From this perspective, it is often emphasized that immigrants are welcome and make important contributions to the communities in which they live. However, in comparison to the positions outlined above, they remain less concrete than the very pictorial representations of right-wing commentators. It was striking that the normative foundations for a pro-migration stance were not very clearly articulated in the material. In August 2023, for instance, the Mayor of Woburn, Scott Galvin, emphasised the inclusive nature of his city when commenting on the placement of migrants in the city’s shelters. Although he explicitly criticizes the political handling of the issue, he states:

“*Woburn has always been a welcoming community and its increasingly diverse population has strengthened our City*.” (Galvin, 2023)

From this perspective, it is frequently argued that immigration and the increasing diversity of the population contribute to the wealth and resilience of the city. The quote exemplifies an overarching theme that recurrently emerges in the discourse, underscoring a stance of openness toward migrants. It is employed as a means of self-description, thereby adopting a distinct moral perspective. While this openness is perceived as a contribution to the community, it is intertwined with the portrayal of U. S. society as a whole. Consequently, a self-image is articulated that explicitly challenges anti-migration positions. The historical intertwining of migration with discourses of Americanness (Friedman, 1991) suggests that emphasizing diversity as a welcomed attribute is a plausible position, and this viewpoint is particularly evident in the societal understanding: welcoming immigrants is not only a typical American value, but also a moral obligation. These perspectives are also associated with constructions that emphasize the inclusive character and significance of migration for American society.

### 5.3 Citizenship as both exclusion and inclusion

The liberal colonial model of citizenship is associated with a distinctive positioning of the individual within the public discourse. The use of pejorative terms such as ‘immigrant’, ‘non-citizen’, and ‘illegal” to describe an individual effectively marks them as an outsider, excluded from the community. In the liberal colonial model of citizenship, the nation-state is responsible for allocating citizenship by birthright (Shachar, 2021 [2009]). This is determined by whether an individual is born on the territory of the United States or if either of their parents is a citizen of the United States (*ius soli and ius sanguinis*). According to the planetary model, citizenship is associated with one’s place of residence (*ius domicilii*). One becomes a citizen by living in a city or State, as this enables one to become a member of the community. These include the right to live in safety, to earn a living, and to have a family. These different positions are reflected in an article in a local newspaper. In an attempt to point out the main lines of argument for and against the inclusion of immigrants, it subsumes them as follows:

“*Build the Wall! Massachusetts’s reputation for generous entitlement benefits is a magnet for migrants coming over the southern border, creating a vicious cycle that leads to more and more government spending and more and more in-migration. The result is an unfair burden on Massachusetts taxpayers*.

*All are Welcome! Regardless of what the federal government or any other state does, Massachusetts should hold itself to a higher moral standard, by doing whatever it takes to find adequate shelter for our homeless families, no matter who they are, where they come from, or how long they’ve been here*” (Common Wealth Beacon, James Peyser, 2024)

The previously mentioned aspects are summarized once again here. The homogenization of immigrants, in conjunction with the fear of scarce resources, leads some to perceive migration as a primarily negative phenomenon, while others emphasize normative standards and equal treatment. These positions, therefore, signify substantial discrepancies in the construction of society, which are subsequently manifested in inquiries concerning the legitimate access to rights. Those who advocate for immigrants’ rights are local governments, non-governmental organisations (NGOs), and support groups, as well as immigrant groups and academics. These positions represent planetary citizenship in different ways (Isin and Nielsen, 2008; Isin, 2017). The prevailing normative values in this context prioritize human rights, social equality, and justice. In contrast to the notion that immigration poses a threat to society, these positions underscore the fundamental right to equal treatment and protection for individuals who seek refuge in other countries. The inclusive society is not the nationally constituted society that is merely connected to the concept of a fixed nation of people that formed their political will in the context of the nation state. Rather, it is one that is constantly produced and reproduced in the interaction of people co-living within a polity, whether that be a city or a state. Planetary citizenship acknowledges the existence of a multifaceted and intricate system of polities (Walby, 2003). It advocates for an inclusive and global justice framework, predicated on the premise that citizenship is a fundamental human right for all individuals residing within a political community.

## 6 Conclusion: Sanctuary cities and the re-negotiation of the migration-citizenship nexus in the United States

This paper posits that discourses concerning the right to social housing can offer insights into the manner in which membership is constructed in American society and the knowledge orders that underlie contemporary debates on migration and citizenship. A distinction was made between positions that emphasize the societal openness towards those in need and those that privilege the rights of national citizens exclusively. The latter group seeks to modify support measures for immigrants in accordance with this privilege on a national basis. An analysis of these positions was conducted to explore the manifestation of diverging orders of knowledge surrounding citizenship, as membership and belonging. Liberal colonial citizenship is characterized by its focus on national citizenship related to the conception of the nation-state as the primary reference point for the allocation of rights. In contrast, planetary citizenship refers to human rights, social justice and solidarity of all human beings, regardless of legal status or nationality, including migrants.

The two aforementioned knowledge orders are indicative of processes of social polarisation and the incompatibility of the values that underlie different conceptions of democracy (Calhoun et al., 2024, 17). The present article has examined these two contrasting models of citizenship that underpin current social conflicts over so-called ‘sanctuary’ laws, which promote the inclusion of immigrants regardless of citizenship status. The discrepancies between planetary citizenship and liberal-colonial citizenship are exemplified in the struggles over the migration-citizenship nexus, as illustrated by the discourses surrounding the Massachusetts right to shelter law, which serves as an illustration and can be regarded as a sanctuary law as it does not currently differentiate between citizens and non-citizens.

An analysis of discursive positions on this issue reveals how different normative orders and associated notions of citizenship are invoked to justify or discredit political practices and access to rights. Consequently, the ongoing debates are indicative of a substantial political polarization that is inherently associated with the conflicting forms of institutionalization. These forms of institutionalization can be understood as the articulation of competing knowledge orders about belonging. The prevailing discourse surrounding sanctuary cities is characterized by the interplay of two conflicting knowledge orders. It is evident that these knowledge orders have a profound influence on the discourse surrounding the distribution of power over migrants, the governance of these populations, and the interpretation of the implications of immigration.

Citizenship in the United States emerges as a multileveled process that is subject to negotiations between federal, state, and local administrative entities. In consequence of the divergent normative orders and concomitant political affiliations, the conception of what citizenship entails varies considerably between different levels of governance. It is not necessarily the politicization of certain areas, such as religion, immigration, or public security, which is called into question. Rather, it is about the different interpretations of the issues in these areas, which are based on narratives about immigrants and social cohesion. These interpretations reveal different perspectives on belonging, which can either legitimize or delegitimize access to rights and can lead to social exclusion or inclusion. While immigration is widely recognized as a phenomenon that has shaped the United States and its society, interpretations of who is welcome, and the extent to which current migration movements represent an opportunity or obstacle to social cohesion, differ substantially. These interpretations often imply racialized orders of belonging.

In this context, the term ‘planetary citizenship’ has been proposed to signify the articulation of one of these knowledge orders, highlighting the need to link discourses on social justice and human rights to the side-effects of capitalism, globalization, and the challenges of late modernity—particularly contemporary migration phenomena. As migrations often originate from economic inequalities, conflicts and environmental crises, they underline the urgent need to rethink citizenship from a global perspective. This would involve promoting rights and responsibilities that transcend national borders and embrace a more inclusive, solidarity-based vision. On a planetary scale the United Nations (United Nations, 2022) has acknowledged the need for safe housing in the context of climate change, thus complementing discussions on the side effects of capitalism and globalization. In this example, the legitimate reasons for leaving a country remain vague. Wars, conflicts, and violence are recurring reasons in human rights discourse, but the question of what constitutes a dignified life remains. Furthermore, the question remains open as to changes in living conditions on the planet can justify granting of rights to migrants. Further research could clarify the factors that contribute to authorising and legitimising migration as well as the normative foundations behind policy decision in sanctuary cities.

Of particular interest is the link these discourses establish between immigration and public safety. While public safety is generally considered desirable, there are different interpretations of the extent to which protecting migrants can lead to exclusionary outcomes. This suggests similar views regarding the legitimacy of political action. Liberal-colonial and planetary citizenship are based on the idea that citizenship is a relationship between a polity and its individuals, complete with associated rights. From the liberal colonial perspective, the only relevant polity is the nation-state, responsible for ensuring each individual’s rights. Planetary citizenship, on the other hand, encompasses a variety of polities, including cities, states and supranational political unions such as the European Union. Individual rights are based on human rights and global justice. While these perspectives provide important points of reference for claiming rights through visions of membership and belonging beyond the nation-state context, it is noteworthy that the nation-state remains the dominant and most powerful agent in granting citizenship rights within the global context of segmentary differentiation into nation-states. The nation-state’s hegemony as a political actor and mode of social inclusion poses risks, particularly when communities fear that powerful national institutions are being implemented against their will. Against this background, the planetary model of citizenship demonstrates how the normative orders of late modernity are translated into political programs at the urban level that transcend the nation-state and fundamentally challenge its approach to migration. These policies continue to provide a basis for creating spaces for coexistence that undermine the processes of boundary drawing that citizenship necessarily entails. At the same time, planetary citizenship moves beyond familiar notions of global citizenship because it redefines the framework of responsibility, refocusing attention on legitimate reasons for migration.

## Data Availability

The raw data supporting the conclusions of this article will be made available by the authors, without undue reservation.

## Footnotes

^1^It can be argued that further ideal types can be identified within this field of tension. Neoliberal citizenship has been suggested as a further development of liberal-colonial citizenship (Joppke, 2021). Furthermore, it is conceivable that current movements towards authoritarianism, as can be observed in the United States, for example, will give rise to new variants of liberal-colonial citizenship or even transformations of basic assumptions (e.g., the relevance of the sovereign nation state for the global order). However, since we believe that there is a need to address colonial continuities within basic positions of the current discourses, we have limited this article to examining the two contrasting perspectives suggested here in detail.

^2^The objective of discourse analysis is to examine the formation of knowledge, the structure of symbolic systems, and the development of subjectivity. The analysis should therefore be distinguished from practices that also adhere to these logics and render certain actions more probable. However, the practice itself pertains to specific actions within a given situation, which are influenced by numerous other situational factors.

^3^This campaign was not part of the narrower evaluation corpus but is cited here to get to the heart of the legal implications of this vision.
