# Supplementary material for: Contesting liberal-colonial citizenship: the planetary model of citizenship and the struggle for the ‘right to shelter’
Source: Front Sociol. 2025 Jul 9;10:1520611. doi: 10.3389/fsoc.2025.1520611 (PMC12285653; doi:10.3389/fsoc.2025.1520611)
Supplement: Supplementary file 1 [file Data_Sheet_1.docx]

**Materials „right to shelter“ in Massachussetts, 3/23-6/24**

Remark: The materials for a detailed analysis were selected according to the principles of grounded theory.

| **No.** | **Date** | **Kind** | **Title/Description** | **Persons/Institutions** |
| --- | --- | --- | --- | --- |
| 1 | 14.12.2023 | article | In New York and Massachusetts, Right to Shelter Is Under Attack | Isaiah Thompson, Nonprofit Quarterly: https://nonprofitquarterly.org/in-new-york-and-massachusetts-right-to-shelter-is-under-attack/  (06.08.2024) |
| 2 | 26.1.2024 | article | Running Out of Room: The Right to Shelter in Massachusetts | Boston University School of Law, Juliana Hubbard: https://sites.bu.edu/dome/2024/01/26/running-out-of-room-the-right-to-shelter-in-massachusetts/ (05.08.2024) |
| 3 | 1.11.2023 | article | Mass. Residents say they support right-to-shelter law, sort of | Commonwealth Beacon, Gintatutas Dumicius:  https://commonwealthbeacon.org/by-the-numbers/mass-residents-say-they-support-right-to-shelter-law-sort-of/ (06.08.2024) |
| 4 | 31.03.2023 | open letter | Letter to Secretary Alejandro Mayorkas (U.S. Department of Homeland Securtity) and Director Ur M. Jadou (U.S. Citizenship and Immigration Services | Elizabeth Warren et al.  https://www.warren.senate.gov/imo/  media/doc/2023.07.31%20Letter%20to%20DHS  %20and%20USCIS%20on%20expediting%20work%20  authorizations%20for%20humanitarian%20parolees1.pdf (5.8.2024) |
| 5 | 03.08.2023 | letter | Letter to Alejandro Mayorkas, Secretary of Homeland Security | Maura T. Healey (Governor): https://www.mass.gov/files/documents/2023/08/08/Emergency%20Declaration%20Letter_0.pdf (5.8.2024) |
| 6 | 16.10.2023 | article | State’s shelter system to run out of capacity in weeks | Emma McCorkindale, WWLP: https://www.wwlp.com/news/state-politics/states-shelter-system-to-run-out-of-capacity-in-weeks/?ipid=promo-link-block1 (5.8.2024) |
| 7 | 16.10.2023 | article | What’s next for right to shelter law in Massachusetts? | WWLP: https://www.wwlp.com/news/state-politics/whats-next-for-right-to-shelter-law-in-massachusetts/ (5.8.2024) |
| 8 | 31.3.2024 | fact sheet | Emergency Assistance (EA) Family Shelter Program in Massachusetts | Executive Office of Housing and Livable Communities: https://www.mass.gov/doc/ea-family-shelter-fact-sheet-faq/download (5.8.2024) |
| 9 | 15.5.2024 | comment | ‘Bleep you! Bleep America!’ Bleep Norfolk | The Boston Herald, Howard Carr: https://www.bostonherald.com/2024/05/15/howie-carr-bleep-you-bleep-america-beep-norfolk/ |
| 10 | 06.09.2023 | petition | An Act ensuring fair housing for homeless families | Rep. Peter Durant: https://malegislature.gov/Bills/193/HD4561 (08.08.2024) |
| 11 | 25.2.2024 | article/analysis | Should we repeal the state’s right-to-shelter-guanrantee? | CommonWealth Beacon: James Peyser (author and former secretary of education in M.A.): https://commonwealthbeacon.org/opinion/should-we-repeal-the-states-right-to-shelter-guarantee/ (5.8.24) |
| 12 | 21.12.2023 | video | State Sen. Durant wants to amend state’s right to shelter law | Spectrum News 1, Amanda Keane: https://spectrumnews1.com/ma/worcester/news/2023/12/21/durant-shelter-capacity (08.08.2024) |
| 13 | 6.10.2023 | video | Senator Fattman on “Right to Shelter” Law” (Hearing in the Senate of the State of Massachusetts ) | Ryan Fattman: https://www.facebook.com/watch/?v=165318979905387 (08.08.2024) |
| 14 | 26.10.2023 (filed) | petition | An Act ensuring fair housing for homeless families | Ryan C. Fattmann, Susan Williams Gifford: https://malegislature.gov/Bills/193/SD2824 (09.08.2023) |
| 15 | 15.05.2024 | radio interview | Assessing MA’s Right to Shelter Law | NightSide with Dan Rea, Peter Durant: https://music.amazon.de/podcasts/3fa02b8f-2461-44ef-951d-aa679f735649/episodes/7557afe0-91b5-41d7-9164-3d073ca8f76b/nightside-with-dan-rea-assessing-ma%E2%80%99s-right-to-shelter-law---part-1 (09.08.2024) |
| 16 | 30.04.2024 | press release | Budget Update: State Legislature Upholds Right to Shelter Law – with temporary limits | Massachusetts Immigrants & Refugee Advocacy Coalition (MIRA): https://miracoalition.org/news/budget-update-state-legislature-upholds-right-to-shelter-law-with-temporary-limits/ (09.08.2024) |
| 17 | 12.06.2024 | article | Massachusetts is latest blue state to enforce migrant shelter stay limits | Politico (Kelly Garrity): https://www.politico.com/news/2024/06/12/massachusetts-to-begin-enforcing-shelter-limits-00163041 (14.08.2024) |
| 18 | 18.08.2023 | press release | Press release by Scott D. Galvin, Mayor of Woburn | Scott Galvin: https://woburnma.gov/wp-content/uploads/2023/08/8-18-23-Galvin-press-release-Right-To-Shelter-law.pdf (14.08.2024) |
| 19 | 12.2.2024 | article | Lawmakers propose changing state’s “Right to Shelter” law | Spectrum News 1 (Amanda Keane): https://spectrumnews1.com/ma/worcester/news/2024/02/12/right-to-shelter-law (27.3.2024) |
| 20 | 19.03.2023 | press release | Woburn Migrant/Refugee Update | Scott D. Galvin (Mayor): https://woburnma.gov/news/2023/09/woburn-migrant-refugee-update/ (15.08.2024) |
